# In-Silico Exploration of Plant Metabolites as Potential Remedies of Norovirus

**DOI:** 10.1155/2022/8905962

**Published:** 2022-10-20

**Authors:** Zenifer Alam, Md. Nazmul Islam Bappy, Abida Sultana, Fayeza Sadia Laskar, Kawsar Miah, Kazi Md. Ali Zinnah, Sudeb Saha

**Affiliations:** ^1^Faculty of Biotechnology and Genetic Engineering, Sylhet Agricultural University, Sylhet-3100, Bangladesh; ^2^Department of Animal and Fish Biotechnology, Sylhet Agricultural University, Sylhet-3100, Bangladesh; ^3^Faculty of Veterinary, Animal and Biomedical Sciences, Sylhet Agricultural University, Sylhet, Bangladesh; ^4^Department of Dairy Science, Sylhet Agricultural University, Sylhet-3100, Bangladesh

## Abstract

Research is still being carried out to develop specific medications or vaccinations to fight norovirus, a key contributor to foodborne illness. This study evaluated certain plant-based active chemicals as prospective candidates for such treatments using virtual screening techniques and other computer assessments. Twenty (20) plant metabolites were tested against the norovirus VP1, VP2, P48, and P22 protein domains using the molecular docking method. In terms of the lowest global binding energy, Asiatic acid, avicularin, guaijaverin, and curcumin exhibited the highest binding affinity with all selected proteins. Each viral protein's essential binding sites with the potential drugs and drug surface hotspots were uncovered. The ADMET (absorption, distribution, metabolism, excretion, and toxicity) analysis was used to further analyze the pharmacological profiles of the top candidates. According to the results, none of the substances showed any adverse consequences that would reduce their drug-like properties. According to the analysis of the toxicity pattern, no detectable tumorigenic, mutagenic, irritating, or reproductive effects of the compounds were discovered. However, among the top four alternatives, curcumin exhibited the highest levels of cytotoxicity and immunotoxicity. These discoveries may open the way for the development of effective norovirus therapies and safety measures. Due to the positive outcomes, we strongly propose more *in vivo* experiments for the experimental validation of our findings.

## 1. Introduction

Noroviruses are a gathering of nonenveloped, single-abandoned, and positive-sense RNA infections that have a place with the norovirus class in the Caliciviridae family [1]. They cause severe outbreaks of disease in confined and semiclosed locations, such as nursing homes, schools, hospitals, military bases, and cruise ships and spread swiftly through contaminated water, food, or surfaces. However, 607 of 680 Nov episodes (89%) were related to individual-to-individual transmission, which oftentimes elaborate unfortunate hand cleanliness, as well as surface-to-surface transmission [2]. They cause 700 million episodes of loose bowels overall every year, representing 20% of all looseness of the bowels cases and causing huge dismalness and mortality [3]. Human noroviruses (Nov) are the top cause of foodborne disease in the United States, accounting for more than 58 percent of cases with known etiologies and 49% of outbreaks of foodborne gastroenteritis [4]. They are the most common viral diseases that cause acute gastroenteritis (AGE) in people of any age in both developed and developing nations. Around 20 million AGE incidences occur in the United States every year, bringing about roughly 70,000 hospitalizations and up to 800 fatalities. The norovirus ailment trouble is most noteworthy in low-and center-pay nations, guaranteeing more than 200,000 lives every year and costing the medical care framework USD 4.2 billion in direct expenses and USD 60.3 billion in friendly financial harm [5] and thus, they pose a serious threat to global public health. Additionally, Nov is to blame for 73% to 95% of all pandemic cases of nonbacterial gastroenteritis worldwide.

Firstly, human noroviruses have changed hereditarily and antigenically, with 35 genotypes separated into five genogroups (GI, GII, GIV, GVIII, and GIX) and different genotype variations are often found simultaneously [6]. New variations foster routinely even inside a solitary genotype, for example, the predominant genogroup II, genotype 4 (GII.4) because of quick advancement. Secondly, noroviruses do not foster well in culture cells and finally, the lack of a standard cell culture-based measure of antibody response, a reliable animal model, and strong immunological correlates of norovirus immunity makes it difficult to compare different vaccine candidates. However, four applicant immunizations are now in the clinical improvement stage [7] and a computer-based vaccine model has been proposed that requires further *in vitro* and *in vivo* trials [8]. Therefore, screening therapeutics against this foe is of high need at the current time.

Antioxidant-rich medicinal plants have the ability to treat serious illnesses such as cancer, Alzheimer's disease, diabetes, malaria, and cardiac conditions while reducing drug toxicity. In a few studies, plant antimicrobials have been tested against both encapsulated and nonencapsulated pathogens. The efficiency of plant antimicrobials against nonenveloped viral contaminations or their substitutes has recently attracted the attention of a small group of researchers. To avoid the negative effects of synthetic pharmaceuticals, the use of natural metabolites against Norovirus as new medications can be a great option. Therefore, the present study aimed to identify plant-based compounds using virtual screening methods and various computational investigations that could be effective against norovirus, as well as safe for human use.

## 2. Materials and Methods

**Figure 1:**
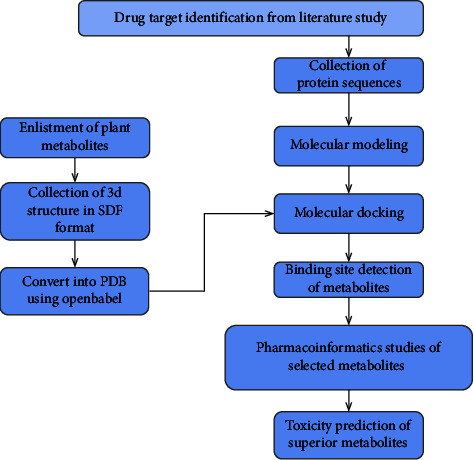
Overview of the protocol.

Different bioinformatics tools were utilized to screen potential drugs against Norovirus (Figure 1). The steps are as follows:

### 2.1. Selection and Collection of Protein Sequences

After reviewing the literature, suitable proteins against which drugs can be designed were identified. Different proteins that were suggested for drug targets of norovirus by earlier research were selected. Then, the National Center for Biotechnology Information Genome database was used to extract the chosen protein sequences (https://www.ncbi.nlm.nih.gov/genome).

### 2.2. Prediction and Refinement of 3D Structures of the Proteins

The Protein Data Bank did not have the chosen proteins' structures (RCSB PDB). As a result, the atomic models of the exceptional proteins were predicted using the RaptorX server (https://raptorx.uchicago.edu/), a bioinformatics method for anticipating a three-layered structure model of protein particles from amino corrosive groupings [9], and then the predicted structures were refined in the GalaxyWEB server https://galaxy.seoklab.org/cg [10]. Then, for each protein, the top model was chosen based on the quality of the ERRAT plot and the Ramachandran plot analysis utilizing the Saves server v6.0 (https://saves.mbi.ucla.edu/). The “general quality element” of the nonfortified nuclear collaborations is produced using the ERRAT plot, which is then used to approve the protein structure. This step is to validate the protein model, which was created using a homology modeler and is dependent on a high score.

### 2.3. Selection and Collection of Metabolites Structure

Twenty metabolites were ultimately chosen for the test as a norovirus treatment after examining the literature on plant metabolites. Then, using the Pubchem database, a total of 20 metabolites in SDF format from various classes were obtained (Table 1). The Open Babel v2.3 program, a chemical toolkit created to speak the various languages of chemical data, was then used to convert the designs into the PDB format [11].

### 2.4. Analysis of the Binding Capacity and Binding Residues of Plant Metabolites Against Selected Proteins

Atomic docking is a fantastic tool that shows how tiny ligands and macromolecules can work together, making it suited for drug disclosure. PatchDock Server was utilized for docking [12], as subatomic docking provides the essential resources to find effective treatments against explicit medication focus of lethal pathogens [13]. Here, the metabolites were designated as the ligand and the proteins as the receptor. The FireDock refining device was then used to polish the docked structures [14]. After that, the PyMOL v2.0 software was used for visualization and to identify the polar binding and nonpolar residues that form a bond with chosen metabolites [15].

### 2.5. Pharmacoinformatics Studies

The four important processes of adsorption, distribution, metabolism, and excretion (ADME) have a substantial impact on drug levels and the energy of drug permeability to tissues within a species. Early ADME assessment during the disclosure stage has been found to significantly reduce the minor amount of pharmacokinetics-related disappointment during the clinical phases [16]. For the prediction of ADME, computer models have been promoted as a viable alternative to trial procedures, particularly at the early stages when there are a variety of examined synthetic designs but limited availability of mixtures [17]. The ADME properties of the top four metabolites were examined using the SwissADME server [17]. The drugs were submitted to the server in the SDF organization, entirely converted to SMILES, and hurried to receive the forecasts after that. The blood-cerebrum obstruction (BBB) in the concentrated compounds was calculated using the BOILED-Egg model [18].

### 2.6. Toxicity Analysis of the Superior Plant Metabolites

Finally, the pkCSM server was utilized for anticipating the toxicity profile of top potential remedies. It is a compelling strategy for anticipating pharmacokinetic properties relying upon diagram-based marks reflecting distance designs between molecules [19].

## 3. Results

### 3.1. Retrieval of Protein Sequences

Four different proteins VP1 (Accession: AII73783.1), VP2 (Accession: AII73784.1), p48 (Accession: YP_009238493.1), and p22 (Accession: YP_009238495.1) were selected as suitable drug targets and their respective sequences were collected from NCBI databases.

### 3.2. Molecular Modeling and Quality Assessment of the Predicted Models

**Figure 2:**
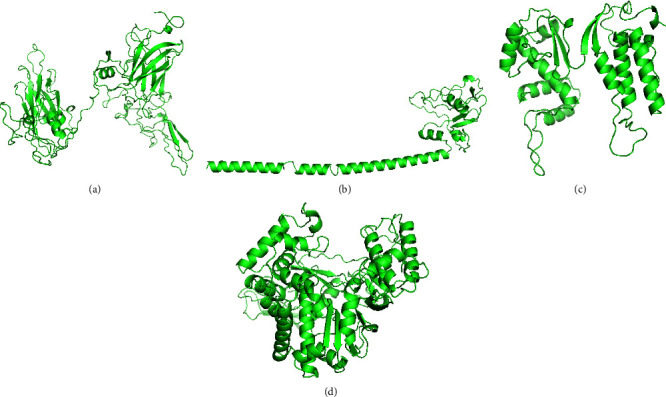
3D structure of (a) VP1; (b) VP2; (c) P48; and (d) P22 proteins.

**Figure 3:**
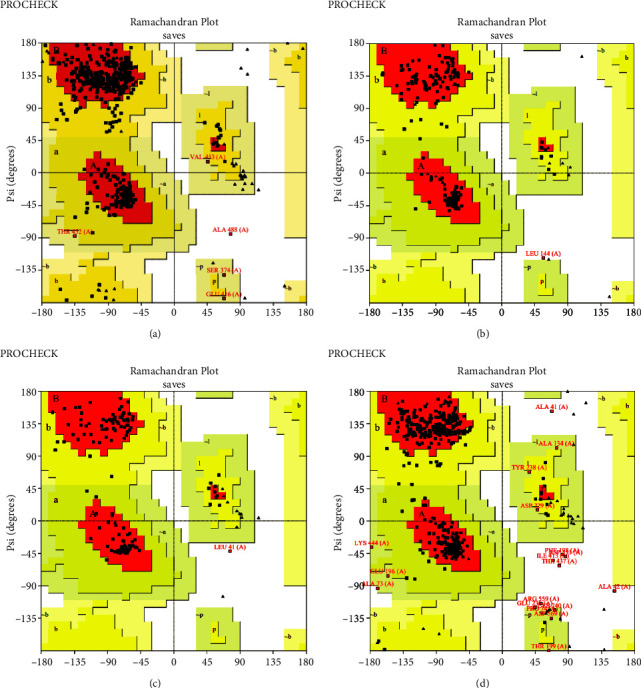
Ramachandran plot of (a) VP1; (b) VP2; (c) P48; and (d) P22 proteins.

The RaptorX server provided five models for each protein. The finest one was then enhanced using the GalaxyWEB server after researching the ERRAT esteem and Ramachandran plot. Based on the results of the Ramachandran plot and ERRAT predicted score, this server provided 10 refined models of each protein, from which the best models of each protein were chosen (Figures 2 and 3 and Table 1).

### 3.3. Enlistment and Collection of Plant Metabolites Structure

A total of 20 metabolites in SDF format from various classes were obtained from the PubChem database using this method (Supplementary file 1). PubChem is a database of chemical compounds and their responses to biological experiments is called PubChem. These metabolites are employed in several investigations, primarily against different viruses and have been experimentally proven to have certain purported health-beneficial effects, such as anticancer, antibacterial, antiviral, and antidiabetic. We use these metabolites because of their qualities and appeal to potential drug prospects (Supplementary file 1).

### 3.4. Analysis of Binding Capacity of Plant Metabolites Against Selected Proteins

**Figure 4:**
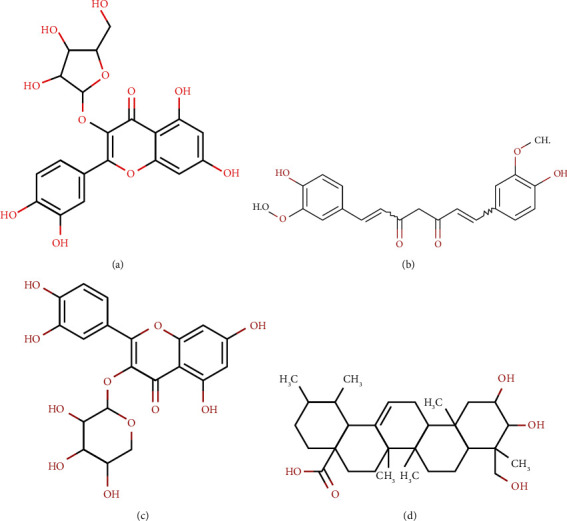
Chemical structures of selected metabolites (a) avicularin, (b) Asiatic acid, (c) curcumin, and (d) guaijaverin.

All the plant metabolites (ligands) docked against all the proteins (macromolecules) (Supplementary file 2). In comparison to all of the chosen proteins, avicularin, Asiatic acid, curcumin, and guaijaverin demonstrated superior global binding energy. The structures of those 4 metabolites are depicted in Figure 4. The metabolites with the highest overall binding energy to the VP1 were discovered to be avicularin, Asiatic acid, curcumin, and guaijaverin correspondingly −59.12 kcal/mol, −50.93 kcal/mol, −54.82 kcal/mol, and −59.12 kcal/mol. Avicularin, Asiatic acid, curcumin, and guaijaverin all had global binding energies of −36.26 kcal/mol, −42.81 kcal/mol, −38.49 kcal/mol, and −30.89 kcal/mol with VP2, respectively. Avicularin, Asiatic acid, curcumin, and guaijaverin all had global binding energies with P48 of −51.06 kcal/mol, −50.93 kcal/mol, −48.79 kcal/mol, and −59.12 kcal/mol, respectively. Avicularin, Asiatic acid, curcumin, and guaijaverin had global binding energies of −41.19 kcal/mol, −51.26 kcal/mol, −50.15 kcal/mol, and −42.08 with P22 respectively (Table 2).

### 3.5. Binding Site Analysis

**Figure 5:**
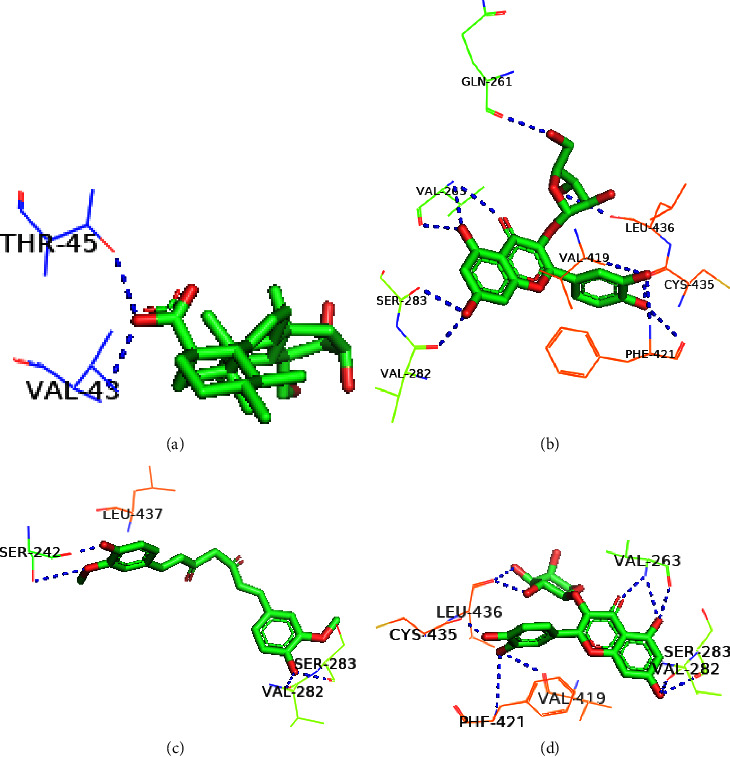
Polar binding sites of VP1 with (a) Asiatic acid, (b) avicularin, (c) curcumin, and (d) guaijaverin.

**Figure 6:**
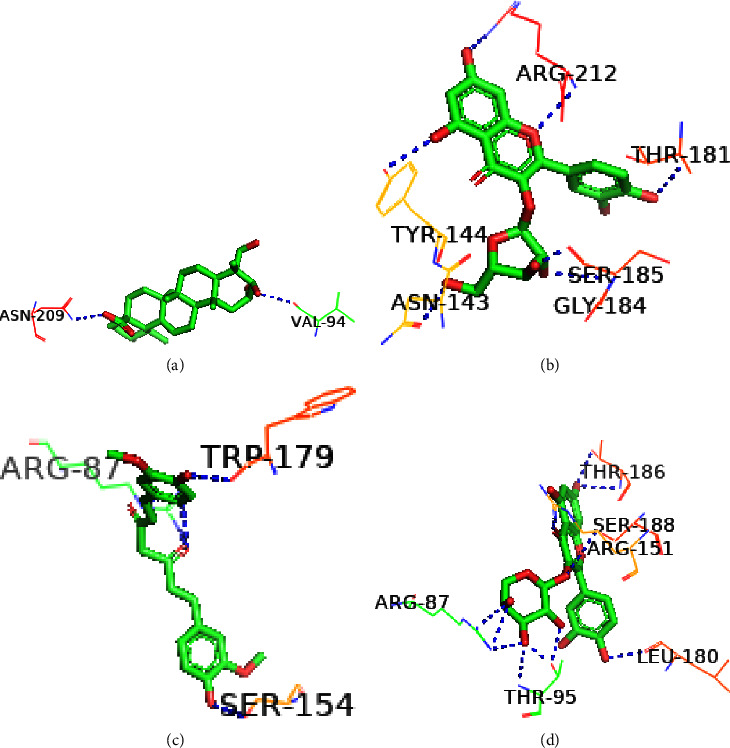
Polar binding sites of VP2 with (a) Asiatic acid, (b) avicularin, (c) curcumin, and (d) guaijaverin.

**Figure 7:**
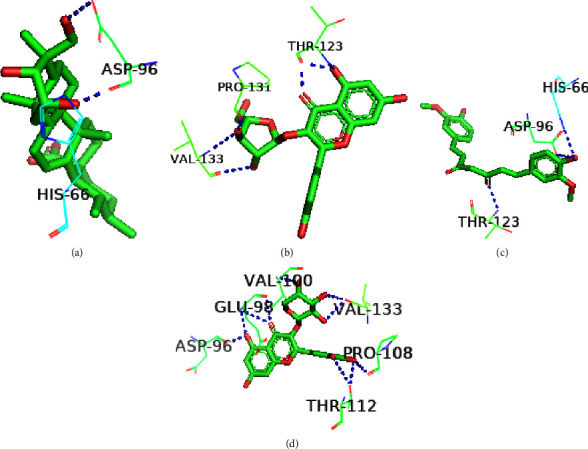
Polar binding sites of P48 with (a) Asiatic acid, (b) avicularin, (c) curcumin, and (d) guaijaverin.

**Figure 8:**
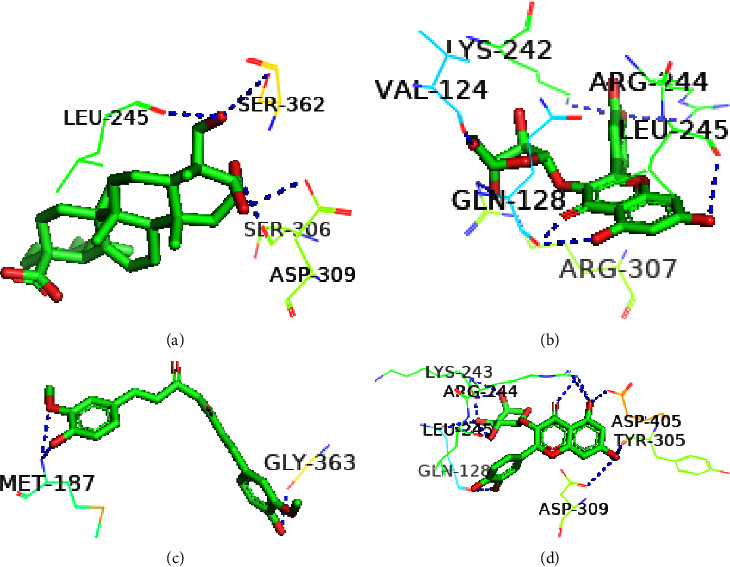
Polar binding sites of P22 with (a) Asiatic acid, (b) avicularin, (c) curcumin, (d) guaijaverin.

The structural conformation of the docked complex was examined to determine the drug surface hotspot of the targeted norovirus proteins. Research has been carried out on the residues interacting with their respective locations and the pattern of ligand binding (Table 2). The 36–49, 260–282, and 419–422 areas of VP1 have a higher binding affinity for the ligands, while Ser283, Val419, and Leu437 were the most prevalent amino acids in interactions with the ligands (Figure 5). The amino acid range 95–186 was the binding hotspot of the norovirus VP2 protein. The docked complexes find the acid binding sites for THR95, ARG87, PRO182, and ILE-97 the most frequent (Figure 6). In the case of the P48 protein, amino acid regions 125–138 were revealed to be the surface hotspots and among the binding residues, PRO125, Val134, and Leu-138 were often observed to interact with the ligands (Figure 7). Amino acids between 111 and 246 of p22 protein were found essential to bind the ligands. The docked complexes find the Gln128, Ser362, and Leu246 residues at maximal times (Figure 8). Figures 5–8 and Table 2 represent all of the polar binding residues, whereas Supplementary File 3 and Table 2 enlisted all of the nonpolar binding residues.

### 3.6. Pharmacoinformatics Studies of Selected Metabolites

**Figure 9:**
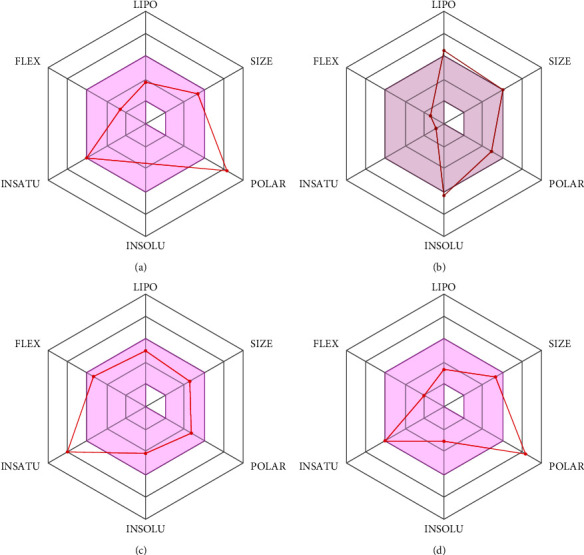
ADME properties of selected metabolites (a) avicularin, (b) Asiatic acid, (c) curcumin, and (d) guaijaverin.

Different ADME qualities, such as physicochemical boundaries, lipophilicity, pharmacokinetics, water dissolvability, and restorative science of top pharmaceuticals were assessed in order to analyze drug profiles of leading antiviral medications (Table 3, Figure 9). Two metabolites, curcumin and asiatic acid, demonstrated substantial gastrointestinal absorption. No blood brain barrier (BBB) permeant was found among the top drugs using the Bubbled Egg model. Restraint effects testing with different CYP isoforms, including CYP1A2, CYP2C9, CYP2C19, CYP2D6, and CYP3A4, revealed that these potent drugs had no chance of interacting with cytochromes P450 isoforms. Every drug displayed weak to significant water solubility. Additionally, guaijaverin and avicularin displayed a single alert for suffering (Table 3).

### 3.7. Toxicity Prediction of Superior Metabolites

Different poisonousness boundaries such as AMES harmfulness, skin refinement, oral rat poisonousness, hepatotoxicity, minnow harmfulness, and so forth were investigated (Table 4). Results uncovered negative results in the AMES test for all medications, which demonstrated the medications as nonmutagenic. As indicated by the outcome, none of the medications went about as hERG I and hERG II inhibitors aside from guaijaverin in hERG II inhibitors. Also, they were predicted to be safe for the skin. LD50, values range from 1.833 to 2.592 mol/kg for these top medications. Minnow toxicity of all medications was more than −0.3 log mM, aside from curcumin, demonstrating them as nonpoisonous. Furthermore, the adverse hepatotoxicity effects of all drugs show that these powerful drugs will not interfere with the liver's normal function.

## 4. Discussion

Noroviruses are the leading cause of foodborne illness worldwide, causing widespread gastroenteritis events that grab the attention of the media and scare both consumers and healthcare professionals. Noroviruses are responsible for about 58% of locally acquired foodborne illnesses, 26% of hospitalizations, and 11% of fatalities. While the overall scope of foodborne illnesses is challenging to manage, it is urgent to develop the skills necessary to identify, manage, and prevent them. Gastroenteritis continues to be a major medical issue that affects people of all ages, but it can be particularly serious for young children, the elderly, and people with impaired immune systems [20].

Every year before they turn five, one million children in nonindustrialized countries still pass away from diarrhea. Of those, 200 000 deaths are thought to have been caused by noroviruses. The development of rotavirus antibodies greatly reduced the overall amount of bowel movements caused by loose stools, however, the fourth and final Millennium Development Goal has not yet been met. In countries where rotavirus vaccination is routinely practiced, noroviruses are currently the primary bacteria causing significant young loose bowels [21]. In the United States, it serves as the basis for one million pediatric clinical consideration visits each year [22].

The previous record for the majority of hospitalizations and fatalities was brought on by gastroenteritis. Despite being misunderstood, norovirus gastroenteritis is undoubtedly linked to an increased risk of hospitalization and mortality in the elderly population and should thus not be considered a minor condition with a short duration [23]. The most well-known locations for norovirus outbreaks are long-term care facilities, followed by cafes, schools, clinics, and ships [24].

Noroviruses are a serious problem at the end of clinic wards [25]. In the UK, over a two-year period, there were about 4000 medical clinic flare-ups that affected 13,000 patients and 3400 staff members and resulted in almost 9000 days of ward closure.

The majority of the time, norovirus symptoms are severe and self-limiting, with 24–48 hours of symptoms before the start of acute regurgitating, queasiness, stomach spasms, myalgias, and exceptionally watery, nonhorrific loose stools that often go away in 2–3 days [26].

However, prolonged and severe illness, including flu-like symptoms, occurs in weak populations (youngsters, older, and immunocompromised). After norovirus gastroenteritis, postirresistible useful gastrointestinal issues, such as cranky inside condition, have been explained. Other specific aftereffects include encephalopathy, spasms, and necrotizing enterocolitis. The location of norovirus in patient serum has barely been identified, but the significance of such results is not yet completely understood. Norovirus gastroenteritis, which is anticipated to occur in 17–18% of immunocompromised individuals, can persist for weeks to years. Undoubtedly, a growing number of studies demonstrate that immunosuppressive therapy increases the risk of norovirus infection [27]. Due to delayed norovirus-related loose stools, these patients typically experience emotional weight loss. This, combined with hunger, dehydration, and an altered GI mucosal border, may increase dejection and interfere with the course of the underlying illness [28].

Natural compounds originating from plants are important because they serve as a model molecule for the creation of potential new drugs. So, in the current investigation, efforts were made to assess several plant-derived metabolites as norovirus inhibitory agents based on their affinity for binding to the chosen specific proteins of the pathogen.

Four drug molecules—curcumin, avicularin, Asiatic acid, and guaijaverin—showed high affinity for each of the four macromolecules, according to the docking results. Due to inadequate ADMET data, many medication development efforts failed during clinical trials. Therefore, whether it is determined by *in vitro*, *in vivo*, or computational methodologies, ADMET data are essential in drug development initiatives.

The top four drug candidates' in-silico ADMET analysis revealed no unfavorable outcomes that would have reduced their drug-likeliness qualities (Table 4). Each drug candidate is GI-absorbable and water-soluble. These four medications will not penetrate the BBB, thus there would be no risk issues. All four medication candidates were found to be noncarcinogenic, nonmutagenic, skin-insensitive, and nonhepatotoxic according to toxicity prediction. Since all drug candidates failed the hERGI and hERG II inhibitors prediction test, they can be considered heart-friendly medications (Table 4). Overall, the toxicity prediction test indicated that those medications are safe to use as norovirus treatment.

## 5. Conclusion

The concept of genomic analysis utilizing several bioinformatics techniques has completely changed the way drugs are discovered. This work could aid in the development of effective therapies against these four distinct proteins with fewer trial-and-error repeat assays, hence saving time and money for performing additional *in vitro* research and assisting in lowering the mortality and morbidity brought on by it. To validate the hypothesis, however, more in vivo tests utilizing model organisms are strongly advised.

## Figures and Tables

**Table 1:** Refined protein model with their ERRAT value and PROCHECK value.

Protein name	ERRAT value	Procheck value	
		Favored region (%)	Disallowed region (%)
VP1	82.366	87.0	0.2
VP2	81.818	92.3	0.5
P48	87.097	92.0	0.4
P22	88.683	88.1	1.5

**Table 2:** Binding sites of avicularin, Asiatic acid, curcumin, and guaijaverin.

Protein name	Active metabolites	Global binding energy	Polar residues	Nonpolar residues
VP1	Avicularin	−59.12	VAL-260, GLN-261,VAL-419, VAL-263, PHE-421, SER-283, VAL-282	GLY-479, LEU-418, PHE-420, LEU-437, PRO-434, PRO-438, SER-262, SER-285, GLN-264, PHE-265, LEU-284
	Asiatic acid	−50.93	VAL-43, THR-45	SER-39, SER-40, ALA-44, ALA-42, THR-41, GLY-38, VAL-49, ALA-37, PRO-35, ASN-50, VAL-36, PRO-S1, LEU-219, TYR-100
	Curcumin	−54.82	VAL-282, SER-283, SER-242	PHE-421, SER-285, VAL-419, LEU-433, LEU-264, LEU-418, PRO-434, CYS-435, LEU-436, TYR-410, SER-244, SER-262, GLN-261, ASN-243, PHE-265, VAL-263, LEU-437, GLN-264, PRO-438
	Guaijaverin	−59.12	PHE-421, VAL-282, CYS-435, SER-283, VAL-419, LEU-436, VAL-263	PHE-265, GLN-264, LEU-437, PRM 24, SER-262, LEU-284, GLY-479, GLN-261, PHE-420, SER-285, HIS-286, LEU-418, VAL-260

VP2	Asiatic acid	−42.81	ASN-209, VAL-94	THR-181, PRO-183, PRO-182, THR-95, SER-136, ARG-211, LEU-180, TRP-179, ARG-96, PRO-108, GLN-135, GLU-109, VAL-134, LEU-206
	Curcumin	−38.49	TRP-155, SER-154, TRP-179, ARG-87	VAL-178, ILE-97, LEU-180, SER-106, THR-95, TRP-155, SER-158, THR-181, PRO-182, ARG-151, SER-188, SER-185, ILE-149, SER-147
	Guaijaverin	−30.89	THR-186, ARG-87, SER-188, ARG-151	VAL-94, ALA-92, PRO-93, SER-106, THR-95, ILE-97, PRO-182, SER-140, TRP-179, SER-185, THR-181, LEU-180, GLY-184, SER-145

P48	Avicularin	−51.06	PRO-131, THR-123, VAL-133	VAL-100, ILE-99, LEU-141, LEU-106, GLU-98, THR-142, PHE-143, VAL-134, LEU-111, PRO-125, LEU-115, THR-112, LEU-132, PRO-122, ARG-124, LEN-120, LYS-121
	Asiatic acid	−50.93	HIS-66, ASP-96	PRO-125, THR-2, THR-123, ARG-124, FHE-14, GLY-95, ASP-97, LYS-121, PRO-131, PRO-122, LEU-141, LEU-120, GLU-98, LEU-133, ILE-99, HIS-66, VAL-134 LEU-115, VAL-100
	Curcumin	−48.79	THR-123, ASP-96, LEU-138, HIS-66	LEU-121, LYS-121, LEU-120, PRO-122, LEU-106, VAL-133, THR-2, ARG-124, VAL-134, GLN-3, VAL-100, GLU-98, ASP-97, LEU-138, SER-101, ILE-99, PHE-143
	Guaijaverin	−59.12	ASP-96, THR-112, GLU-98, VAL-133, PRO-108, VAL-100	LEU-141, THR-123, PHE-143, LEU-132, ASP-97, PRO-122, PRO-131, LYS-121, SER-135, VAL-134, ILE-99, GLU-109, LEU-120, HIS-66, LEU-115, LEU-111

P22	Avicularin	−41.19	LYS-242, ARG-307, ARG-244, LEU-245, SER-310, GLN-128, VAL-124	TRP-247, LEU-246, GLU-127, LEU-129, LYS-243, ILE-241, SER-362, TYR-236, PHE-132, PRO-131, SER-306
	Asiatic acid	−51.26	SER-306, ASP-309, SER-362, LEU-245	PHE-132, SER-310, PRO-131, ARG-307, LEU-129, GLN-128, GLU-127, LYS-242, LEU-246, ARG-244, ILE-241, VAL-124, LYS-243, TYR-238
	Curcumin	−50.15	GLY-363, MET-187	LYS-188, MET-254, PRO-365, THR-179, HIS-185, SER-181, VAL-364, HIS-186, SER-180, SER-249, GLY-182, PRO-184, GLY-248, HIS-183, TRP-247, LEU-246, THR-224, ALA-226, ALA-225
	Guaijaverin	−42.08	GLN-128, LYS-243, LEU-245, ARG-244, ASP-309, TYR-305, ASP-405	SER-362, TRP-247, HIS-375, LEU-246, ASP-304, SER-306, RP-308, SER-310, ARG-307, LYS-242, LEU-129, PHE-132

**Table 3:** SwissADME properties of top metabolites.

Parameter	Features	Metabolites			
Physicochemical parameters	Formula	Avicularin	Asiatic acid	Curcumin	Guaijaverin

	C_20_H_18_O_11_	C_30_H_48_O_5_	C_21_H_20_O_6_	C_20_H_18_O_11_
	Molecular weight gm/mole	434.35 g/mol	488.70 g/mol	368.38 g/mol	434.35 g/mol
	Num. heavy atoms	31	35	27	31
	Num. H-bond acceptors	11	5	6	11
	Num. H-bond donors	7	4	2	7
	Molar refractivity	104.19	139.24	102.80	104.19
TPSA	190.28 Å^2^	97.99 Å^2^	93.06 Å^2^	190.28 Å^2^

Lipophilicity	Log *P*_o/w_ (iLOGP)	1.86	3.20	3.27	1.61

	Log *P*_o/w_ (XLOGP3)	0.98	5.70	3.20	0.43
	Log *P*_o/w_ (WLOGP)	0.10	5.03	3.15	0.10
	Log *P*_o/w_ (MLOGP)	−2.06	4.14	1.47	−2.06
	Log *P*_o/w_ (SILICOS-IT)	0.06	3.96	4.04	−0.10
Consensus log *P*_o/w_	0.19	4.41	3.03	−0.00

Pharmacokinetics	GI absorption	Low	High	High	Low

	BBB permeant	No	No	No	No
	P-gp substrate	No	Yes	No	No
	CYP1A2 inhibitor	No	No	No	No
	CYP2C19 inhibitor	No	No	No	No
	CYP2C9 inhibitor	No	No	Yes	No
	CYP2D6 inhibitor	No	No	No	No
	CYP3A4 inhibitor	No	No	Yes	No
Log *K*_p_ (skin permeation) (cm/s)	−8.25 cm/s	−5.23 cm/s	−6.28 cm/s	−8.64 cm/s

Water solubility	Log *S* (ESOL)	−3.27	−6.33	−3.94	−2.99

	Solubility (mg/ml)	2.34*e*−01 mg/ml	2.29*e*−04 mg/ml	4.22*e*−02 mg/ml	4.47*e*−01 mg/ml
	Solubility (mol/l)	5.39*e*−04 mol/l	4.69*e*−07 mol/l	1.15*e*−04 mol/l	1.03*e*−03 mol/l
	Class	Soluble	Poorly soluble	Soluble	Soluble
	Log *S* (SILICOS-IT)	−2.07	−4.28	−4.83	−3.99
	Solubility (mg/ml)	3.71*e* + 00 mg/ml	2.59*e*−02 mg/ml	5.50*e*−03 mg/ml	4.41*e*−02 mg/ml
	Solubility (mol/l)	8.55*e*−03 mol/l	5.31*e*−05 mol/l	1.49*e*−05 mol/l	1.02*e*−04 mol/l
Class	Soluble	Moderately soluble	Moderately soluble	Soluble

Medicinal chemistry	Pains	1 alert: catechol_A	0 alert	0 alert	1 alert: catechol_A
	Brenk	1 alert: catechol	1 alert: isolated_alkene	2 alerts: beta_keto_anhydride, michael_acceptor_1	1 alert: Catechol
	Leadlikeness	No; 1 violation: MW > 350	No; 2 violations: MW > 350, XLOGP3 > 3.5	No; 2 violations: MW > 350, Rotors > 7	No; 1 violation: MW > 350
	Synthetic accessibility	5.04	6.56	2.97	5.05

**Table 4:** Toxicity parameter of selected chemicals.

Toxicity parameter	Metabolites name			
	Avicularin	Asiatic acid	Curcumin	Guaijaverin
AMES toxicity	No	No	No	No
Max. Tolerated dose (log mg/kg/day)	0.576	0.078	0.081	0.494
hERG I inhibitor	No	No	No	No
hERG II inhibitors	No	No	No	Yes
Oral rat acute toxicity, LD50 (mol/kg)	2.543	2.592	1.833	2.585
Hepatotoxicity	No	No	No	No
Skin sensitisation	No	No	No	No
Minnow toxicity (log mM)	6.668	1.106	−0.081	5.071

## Data Availability

Dataset of this study is available from the corresponding author on reasonable request.

## Supplementary Materials

Supplementary Materials Supplementary file-1: List of plant metabolites used in the study with respective source and activities. Supplementary file-2: Docking results. Supplementary file-3: Nonpolar binding sites of the selected metabolites.
