# Supplementary material for: In-Silico Exploration of Plant Metabolites as Potential Remedies of Norovirus
Source: Adv Virol. 2022 Oct 20;2022:8905962. doi: 10.1155/2022/8905962 (PMC9613402; doi:10.1155/2022/8905962)
Supplement: Supplementary Materials — Supplementary file-1: List of plant metabolites used in the study with respective source and activities. Supplementary file-2: Docking results. Supplementary file-3: Nonpolar binding sites of the selected metabolites. [file 8905962.f1.zip › Supplementary file 1 (1).docx]

**Supplementary file 1**: List of plant metabolites used in the study with respective source and activities

| **Metabolites** | **PubChem CID** | **Source** | **Activities** | **References** |
| --- | --- | --- | --- | --- |
| Allicin | 65036 | Allium sativum | Antimicrorial, antiviral Antioxidant, anti-cancer activity | (Sharma et al.,  2018) |
| Andrographolide | 5318517 | Andrographis paniculata | antioxidant, anti-inflammatory, and anti-cancer | (El-Saber et al.,2020) |
| Apigenin | 5280443 | Vegetable and fruit | Effective in cancer, depression, diabetes & Alzheimer's disease, | (Mussard et al.,2019) |
| Asiatic acid | 119034 | Centella asiatica | Antioxidant, cardioprotective, anti-inflammatory, antitumor, neuroprotective, antimicrobial | (Salehi et al.,2019) |
| Avicularin | 5490064 | Psidium guyava, Lespedeza cuneata | anti-inflammatory, anti-oxidant, hepatoprotective activity | (Meeran et al.,2018) |
| Capsaicin | 1548943 | Capsicum genus | Pruritis, pain relief, non-steroidal anti-inflammatory drug induced gastritis | (Wang et al.,2019) |
| Chavibetol | 596375 | Piper betle | immunomodulatory, radical scavenging | (Hayman et al.,2019) |
| Cinnamic acid | 444539 | Cinnamomum species | Antibacterial; antifungal; antimalarial; antitubercular | (Bhalerao et al.,2013) |
| Curcumin | 969516 | Curcuma longa | antibacterial, anti-inflammatory antiviral, antioxidant, anti- arthritis & anti-cancer activity | (Alsamydai et al.,  2018) |
| Eugenol | 3314 | Ocimum tenuiflorum, Eugenia caryophyllata | antimicrobial, anti-inflammatory, analgesic and antioxidant | (Yamani et al.,  2016) |
| Arjunone | 14034821 | Terminalia arjuna | Arjunone and other compounds have role in antioxidant, antiatherogenic, anti-inflammatory, anti-carcinogenic activity | (Guzman . et al.,2014) |
| Galangin | 5281616 | Honey, Alpinia officinarum, propolis | Anti-cancer, anti-mutagenic, anti-oxidative, radical scavenging etc. | (Al-Samydai et al.,2018) |
| Gentisic acid | 3469 | Gentiana, Citrus, H. rosa- sinensis,O. europaea, S. indicum | Antioxidant, neuroprotective, antiinflammatory, hepatoprotective, antimicrobial activities | Nejad et al.,2017 |
| Guajaverin | 5481224 | Psidium guyava | Anti-plaque activity | (Amalraj et al.,2016) |
| Kaempferol | 5280863 | Vegetable and fruit | Anti-inflammatory, antioxidant, antimicrobial, antitumor, cardioprotective, and antidiabetic activities | (Patel et al.,2012) |
| Luteolin | 5280445 | Carrots, celery peppers, olive peppermint | Anticancer, antioxidant, antimicrobial, anti-inflammatory, and activities | (Abedi et al.,2019) |
| Piperic acid | 5370536 | Piper nigrum | No known function | (Källberg et al.,2012) |
| Polyphenols | 5280343 | Vaccinium sect. Cyanococcus | Antioxidant potential | He et al.,2019 |
| Citrus oil | [440917](https://pubchem.ncbi.nlm.nih.gov/compound/440917) | Citrus sinensis | bio-functional compounds with various health properties, including antioxidant, antimicrobial, anti-inammatory, and cytoprotective activi-ties | Ademosun et al.,2016 |
| Carvacol | 10364 | Origanum  vulgare | Natural antibiotic, | (Gilling et al.,  2014) |
