# Supplementary material for: In-Silico Exploration of Plant Metabolites as Potential Remedies of Norovirus
Source: Adv Virol. 2022 Oct 20;2022:8905962. doi: 10.1155/2022/8905962 (PMC9613402; doi:10.1155/2022/8905962)
Supplement: Supplementary Materials — Supplementary file-1: List of plant metabolites used in the study with respective source and activities. Supplementary file-2: Docking results. Supplementary file-3: Nonpolar binding sites of the selected metabolites. [file 8905962.f1.zip › Supplementary file 2.docx]

**Table: Docking results**

| Protein | Name of Metabolites | Global Binding Energy | ACE | Score | Area |
| --- | --- | --- | --- | --- | --- |
| VP1 | Kaempferol | -62.88 | -21.16 | 5530 | 665.50 |
|  | Pipericacid | -41.31 | -12.50 | 3822 | 446.50 |
|  | Gentisicacid | -40.27 | -12.56 | 4352 | 495.20 |
|  | Cinnamicacid | -33.31 | -9.82 | 3002 | 340.70 |
|  | Allicin | -31.35 | -31.35 | 3368 | 376.60 |
|  | polyphenols | -54.95 | -17.30 | 5136 | 646.60 |
|  | citrusoil | -29.19 | -8.79 | 3152 | 366.30 |
|  | carvacrol | -27.59 | -9.17 | 3172 | 344.60 |
|  | Andrographolide | -47.70 | -15.25 | 5046 | 573.00 |
|  | Apigenin | -45.40 | -14.08 | 4196 | 478.40 |
|  | Capsaicin | -48.55 | -15.69 | 4818 | 608.60 |
|  | Avicularin | -60.41 | -20.02 | 5430 | 662.00 |
|  | Chavibetol | -38.75 | -10.95 | 3582 | 393.60 |
|  | Asiaticacid | -50.93 | -15.66 | 5976 | 674.00 |
|  | Curcumin | -54.82 | -17.38 | 5254 | 627.30 |
|  | Eugenol | -33.08 | -9.72 | 3540 | 401.20 |
|  | Galangin | -44.88 | -14.00 | 4414 | 489.80 |
|  | Arjunone | -46.95 | -15.90 | 5518 | 639.50 |
|  | Luteolin | -44.83 | -13.57 | 4298 | 495.10 |
|  | Guajaverin | -59.12 | -18.92 | 5272 | 663.10 |
| VP2 | Allicin | -19.29 | -6.96 | 2880 | 312.60 |
|  | polyphenols | -38.89 | -11.39 | 4614 | 531.80 |
|  | carvacrol | -20.50 | -5.34 | 2932 | 310.40 |
|  | Andrographolide | -32.73 | -8.66 | 4422 | 539.70 |
|  | Asiaticacid | -42.81 | -13.21 | 5240 | 594.60 |
|  | Curcumin | -38.49 | -7.91 | 4752 | 519.40 |
|  | Eugenol | -25.23 | -8.08 | 3074 | 326.40 |
|  | Guajaverin | -30.89 | -6.92 | 4456 | 517.70 |
|  | Luteolin | -33.61 | -9.10 | 3786 | 418.90 |
|  | Andrographolide | -32.73 | -8.66 | 4422 | 539.70 |
|  | Apigenin | -34.67 | -11.71 | 3720 | 408.10 |
|  | Pipericacid | -25.07 | -7.48 | 3134 | 388.30 |
|  | citrus oil | -22.43 | -6.69 | 2996 | 317.10 |
|  | Arjunone | -33.47 | -11.52 | 4584 | 550.20 |
|  | Galangin | -32.11 | -10.23 | 3792 | 422.10 |
|  | Kaempferol | -39.53 | -9.92 | 4900 | 582.10 |
|  | Gentisicacid | -27.79 | -7.11 | 4012 | 433.90 |
|  | Avicularin | -36.26 | -9.41 | 4468 | 512.10 |
|  | Capsaicin | -28.95 | -8.63 | 4460 | 484.40 |
|  | Chavibetol | -22.46 | -7.24 | 3216 | 347.50 |
| p48 | Pipericacid | -33.06 | -9.43 | 3736 | 412.20 |
|  | Avicularin | -51.06 | -14.33 | 5076 | 627.90 |
|  | citrusoil | -25.03 | -7.58 | 3510 | 382.40 |
|  | Asiaticacid | -50.93 | -15.66 | 4850 | 604.40 |
|  | Allicin | -30.03 | 11.35 | 3332 | 374.60 |
|  | polyphenols | -55.32 | -15.23 | 4608 | 622.90 |
|  | carvacrol | -27.94 | -7.82 | 3534 | 372.90 |
|  | Apigenin | -41.57 | -11.82 | 4122 | 456.90 |
|  | Capsaicin | -37.91 | -13.02 | 4282 | 500.80 |
|  | Chavibetol | -26.54 | -7.29 | 3576 | 391.40 |
|  | Cinnamicacid | -29.86 | -8.26 | 3180 | 340.50 |
|  | Curcumin | -48.79 | -12.35 | 5296 | 616.30 |
|  | Eugenol | -30.12 | -9.01 | 3634 | 391.70 |
|  | Arjunone | -44.68 | -12.56 | 5158 | 597.70 |
|  | Galangin | -44.75 | -12.31 | 4184 | 460.00 |
|  | Gentisicacid | -40.67 | -9.96 | 4382 | 502.40 |
|  | Guajaverin | -59.12 | -18.92 | 4850 | 604.40 |
|  | Kaempferol | -50.47 | -12.46 | 5262 | 633.60 |
|  | Luteolin | -43.81 | -11.56 | 4136 | 476.30 |
|  | Andrographolide | -46.67 | -12.21 | 4640 | 566.40 |
| p22 | Apigenin | -36.83 | -10.99 | 4342 | 485.60 |
|  | Eugenol | -25.83 | -7.73 | 3478 | 389.40 |
|  | polyphenols | -35.17 | -9.21 | 5184 | 584.30 |
|  | Pipericacid | -39.82 | -10.81 | 3848 | 441.20 |
|  | citrusoil | -28.40 | -9.21 | 3238 | 349.90 |
|  | Andrographolide | -38.39 | -9.16 | 5264 | 609.60 |
|  | Avicularin | -41.19 | -8.00 | 5464 | 644.90 |
|  | Chavibetol | -26.65 | -8.32 | 3496 | 391.30 |
|  | Cinnamicacid | -31.77 | -9.21 | 2940 | 317.70 |
|  | Curcumin | -50.15 | -14.84 | 5698 | 660.00 |
|  | Arjunone | -36.66 | -5.35 | 5350 | 623.70 |
|  | Galangin | -41.17 | -11.31 | 4238 | 463.20 |
|  | Kaempferol | -33.70 | -7.70 | 5678 | 652.60 |
|  | Guajaverin | -42.08 | -8.03 | 5306 | 619.70 |
|  | Luteolin | -40.62 | -12.77 | 4314 | 492.70 |
|  | Allicin | -30.04 | -10.22 | 3170 | 363.20 |
|  | carvacrol | -26.13 | -8.68 | 3326 | 367.20 |
|  | Capsaicin | -37.46 | -7.82 | 5112 | 582.70 |
|  | Asiaticacid | -51.26 | -10.53 | 6038 | 780.40 |
|  | Gentisicacid | -33.21 | -5.66 | 4224 | 489.30 |
