# Supplementary material for: In-Silico Exploration of Plant Metabolites as Potential Remedies of Norovirus
Source: Adv Virol. 2022 Oct 20;2022:8905962. doi: 10.1155/2022/8905962 (PMC9613402; doi:10.1155/2022/8905962)
Supplement: Supplementary Materials — Supplementary file-1: List of plant metabolites used in the study with respective source and activities. Supplementary file-2: Docking results. Supplementary file-3: Nonpolar binding sites of the selected metabolites. [file 8905962.f1.zip › Supplementary file 3.docx]

**Vp1 Asiaticacid Nonpolar**

**
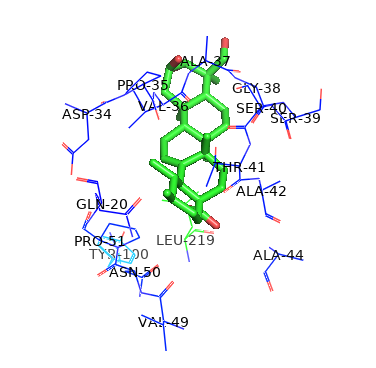
**

**Vp1 Avicularin Nonpolar**

**
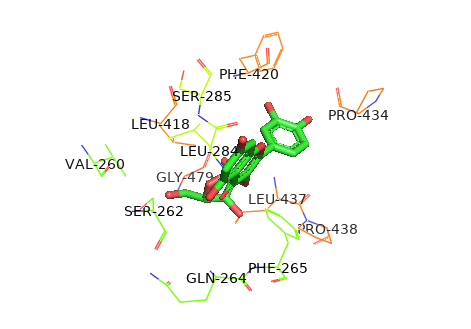
**

**Vp1 Curcumin Nonpolar**

**
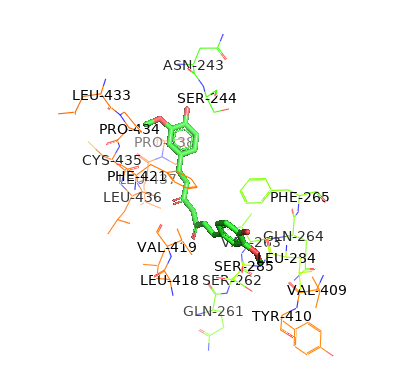
**

**Vp1 Guajaverin Nonpolar**

**
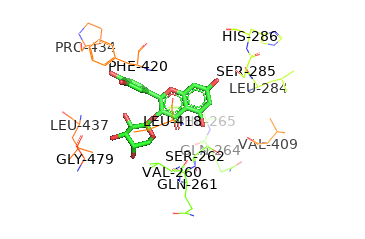
**

**Vp2 Asiaticacid Nonpolar**

**
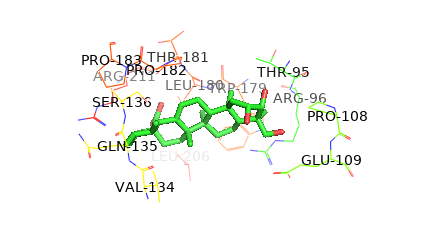
**

**Vp2 Avicularin Nonpolar**

**
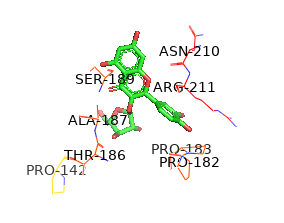
**

**Vp2 Curcumin Nonpolar**

**
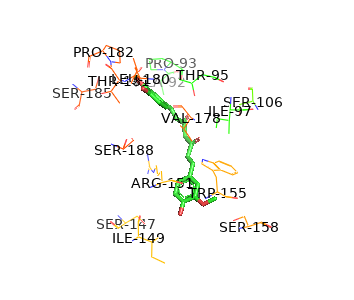
**

**Vp2 Guajaverin Nonpolar**

**
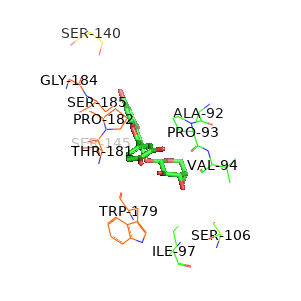
**

**P48 Asiaticacid Nonpolar**

**
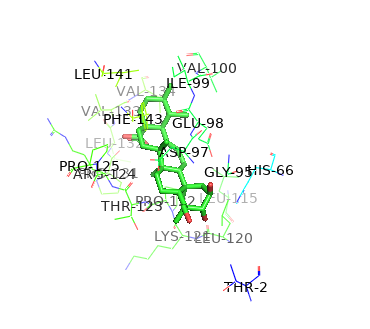
**

**P48 Avicularin Nonpolar**

**
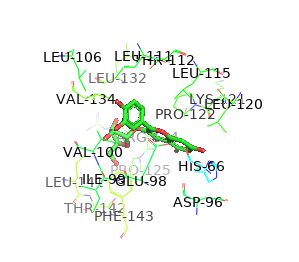
**

**P48 Curcumin Nonpolar**

**
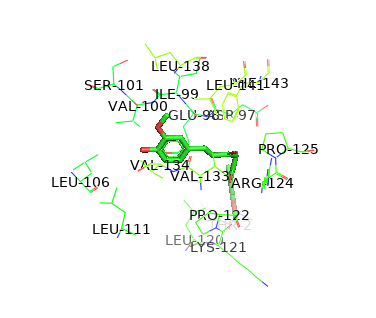
**

**P48 Guajaverin Nonpolar**

**
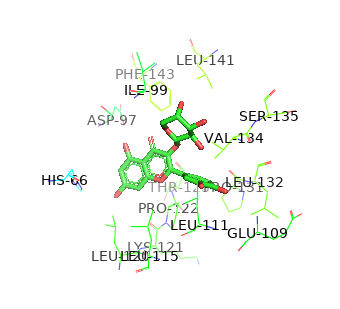
**

**P22 Asiaticacid Nonpolar**

**
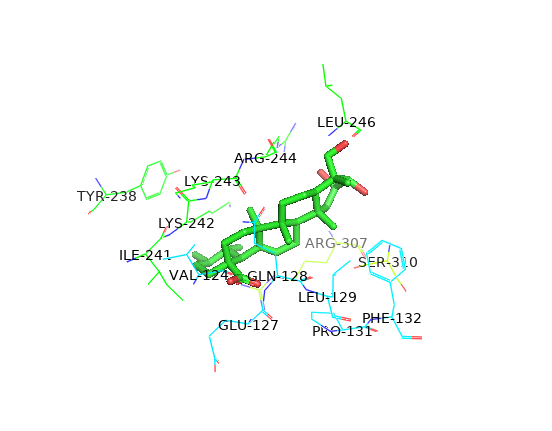
**

**P22 Avicularin Nonpolar**

**
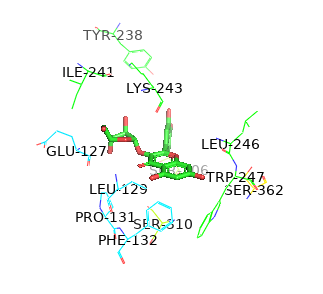
**

**P22 Curcumin Nonpolar**

**
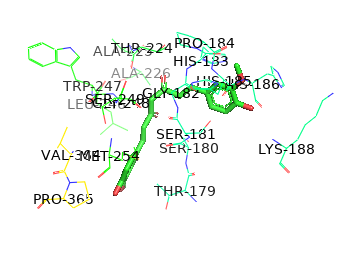
**

**P22 Guajaverin Nonpolar**

**
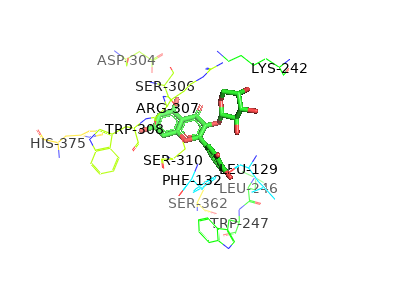
**
